# Development of an optimized tetracycline-inducible expression system to increase the accumulation of interleukin-10 in tobacco BY-2 suspension cells

**DOI:** 10.1186/1472-6750-12-40

**Published:** 2012-07-11

**Authors:** Luisa Bortesi, Thomas Rademacher, Andreas Schiermeyer, Flora Schuster, Mario Pezzotti, Stefan Schillberg

**Affiliations:** 1 Department of Biotechnology, University of Verona, Strada Le Grazie 15, 37134, Verona, Italy; 2 Fraunhofer Institute for Molecular Biology and Applied Ecology (IME), Forckenbeckstrasse 6, 52074, Aachen, Germany; 3 Present address: Institute for Molecular Biotechnology, RWTH Aachen University, Worringerweg 1, 52074, Aachen, Germany

## Abstract

**Background:**

Plant cell suspension cultures can be used for the production of valuable pharmaceutical and industrial proteins. When the recombinant protein is secreted into the culture medium, restricting expression to a defined growth phase can improve both the quality and quantity of the recovered product by minimizing proteolytic activity. Temporal restriction is also useful for recombinant proteins whose constitutive expression affects cell growth and viability, such as viral interleukin-10 (vIL-10).

**Results:**

We have developed a novel, tetracycline-inducible system suitable for tobacco BY-2 suspension cells which increases the yields of vIL-10. The new system is based on a binary vector that is easier to handle than conventional vectors, contains an enhanced inducible promoter and 5′-UTR to improve yields, and incorporates a constitutively-expressed visible marker gene to allow the rapid and straightforward selection of the most promising transformed clones. Stable transformation of BY-2 cells with this vector, without extensive optimization of the induction conditions, led to a 3.5 fold increase in vIL-10 levels compared to constitutive expression in the same host.

**Conclusions:**

We have developed an effective and straightforward molecular farming platform technology that improves both the quality and the quantity of recombinant proteins produced in plant cells, particularly those whose constitutive expression has a negative impact on plant growth and development. Although we tested the platform using vIL-10 produced in BY-2 cells, it can be applied to other host/product combinations and is also useful for basic research requiring strictly controlled transgene expression.

## Background

Molecular farming is the production of valuable pharmaceutical or industrial proteins in whole plants and plant-based systems such as cell suspension cultures [1-6]. Inducible promoters are often used to restrict transgene expression to particular developmental stages in whole plants, or to particular growth phases in cultivated cells, because this can improve product quality and quantity. In cell suspension cultures, it is beneficial to produce secreted recombinant proteins during exponential growth because the beginning of the stationary phase is usually accompanied by the release of proteases that degrade secreted proteins [7,8]. Ideally, bioprocess conditions that minimize proteolysis should be identified and transgene expression should be restricted to the most productive growth phase to prevent extensive protein degradation and loss of activity [9]. Another important justification for the temporal restriction of transgene expression is the tendency for some proteins to inhibit growth and development of the host cells when expressed constitutively, thus reducing yields. For example, if the recombinant protein is an enzyme, its activity can alter cellular metabolism leading to severe pleiotropic effects [10] or it can limit the amount of recombinant protein that the host can produce [11]. Recombinant proteins can also physically interfere with host cellular components leading to highly detrimental or even lethal phenotypes [12,13].

We recently reported the expression of the viral anti-inflammatory cytokine interleukin-10 (vIL-10) in transgenic tobacco plants, and found that retention of the recombinant protein in the endoplasmic reticulum (ER) resulted in stunted plant growth, with the severity of the phenotype determined by the level of protein accumulation [14] and Additional file 1: Figure S1]. Subsequent transient expression experiments have shown that vIL-10 is harmful to the plant not only when it is contained within the ER, but also in the cytosol or following secretion to the apoplast, both of which led to rapid necrosis of the infiltrated leaf area (unpublished data). Even plastid transformation, which is known to protect plants from the toxic effects of some recombinant proteins, produced transplastomic plants with a severe mutant phenotype and low levels of recombinant protein (unpublished data). We therefore decided to express vIL-10 in the tobacco cell line Bright Yellow 2 (BY-2) and to develop strategies based on temporally-restricted inducible expression to avoid the deleterious effects observed *in planta*.

Conditional plant expression systems have been developed using elements from both prokaryotic and eukaryotic promoters. Most rely on chemical rather than physical induction, and the strategy employed is based either on transcriptional repression/activation or post-translational sequestration/release [15]. Some of these systems have been applied successfully to plant suspension cultures [16]. We chose a tetracycline-inducible/derepressible system based on a modified *Cauliflower mosaic virus* 35 S promoter (Triple Op), in which three operator sequences (*tet*O) from the *Escherichia coli* tetracycline operon surround the TATA box [17]. The *tet*O sequences are recognized by the tetracycline repressor (TetR), but its specific binding activity is abolished in the presence of tetracycline or an analog such as doxycycline. In transgenic plants that express TetR constitutively, the Triple Op promoter can therefore be derepressed by supplying tetracycline or doxycycline [15,18]. This system has already been shown to regulate transgene expression precisely in BY-2 cells, including genes encoding β-glucuronidase, green fluorescent protein and auxin binding protein 1 [17,19].

We have developed an improved tetracycline-inducible system that is easy to handle, produces high yields of recombinant protein and that includes a constitutively-expressed visible marker allowing the best-performing clones to be selected rapidly. Stable transformation of BY-2 cells with this vector resulted in the production of 3.5-fold higher levels of vIL-10 compared to constitutive expression in the same host.

## Results

### Constitutive expression of murine and viral IL-10 in BY-2 cells

BY-2 suspension cell lines constitutively expressing viral interleukin 10 (vIL-10) were produced by transforming wild-type cells with construct pTRAkt_ER-vIL-10, which encodes an ER-targeted version of the protein under the transcriptional control of the double enhanced CaMV 35 S promoter [14]. Although transgenic tobacco plants expressing this construct were stunted, those expressing an ER-targeted version of murine IL-10 did not show an obvious morphological phenotype [14]. We therefore produced BY-2 lines constitutively expressing murine IL-10 as a control. These lines were generated by transforming wild-type cells with construct pTRAkt_ER-mIL-10, which is directly analogous to pTRAkt_ER-vIL-10 and contains the same regulatory elements.

We screened 60 independent transgenic callus clones for each construct by immunodotblot analysis to identify the seven best-performing lines. These were tested by ELISA to determine the precise levels of IL-10, and the best performing clone for each construct was used to establish liquid suspension cell cultures. We used the same ELISA to determine the levels of IL-10 in cell pellets from each culture prepared 3, 5 and 7 days after subculture into fresh medium (days post-subculture, dps). The highest yields were observed 5 dps in both lines: 9.3 ± 1.4 μg/g fresh weight (FW) for mIL-10 (range = 2.4-9.3 μg/gFW of callus; average = 5.0; s.d. = 2.8) and 1.4 ± 0.4 μg/g FW for vIL-10 (range = 0.4-1.4 μg/g FW of callus; average 0.8, s.d. = 0.3). Less than 0.2% of each protein was detected in the culture supernatant, as expected for a protein targeted for retention in the ER. The recombinant proteins were of the correct size and mIL-10 was glycosylated as expected (Additional file 2: Figure S2). There was no significant difference in the growth properties of the cell suspension cultures based on pellet weight, but it was clear that vIL-10 was produced at much lower levels than mIL-10. We proposed that vIL-10 was toxic to plant cells, based on its effect on transgenic plants, and that this toxicity had prevented the recovery of high-yielding transgenic BY-2 lines. We therefore investigated the temporal restriction of vIL-10 production using a tetracycline-inducible promoter.

### Generation of a BY-2-TetR line

**Figure 1:**
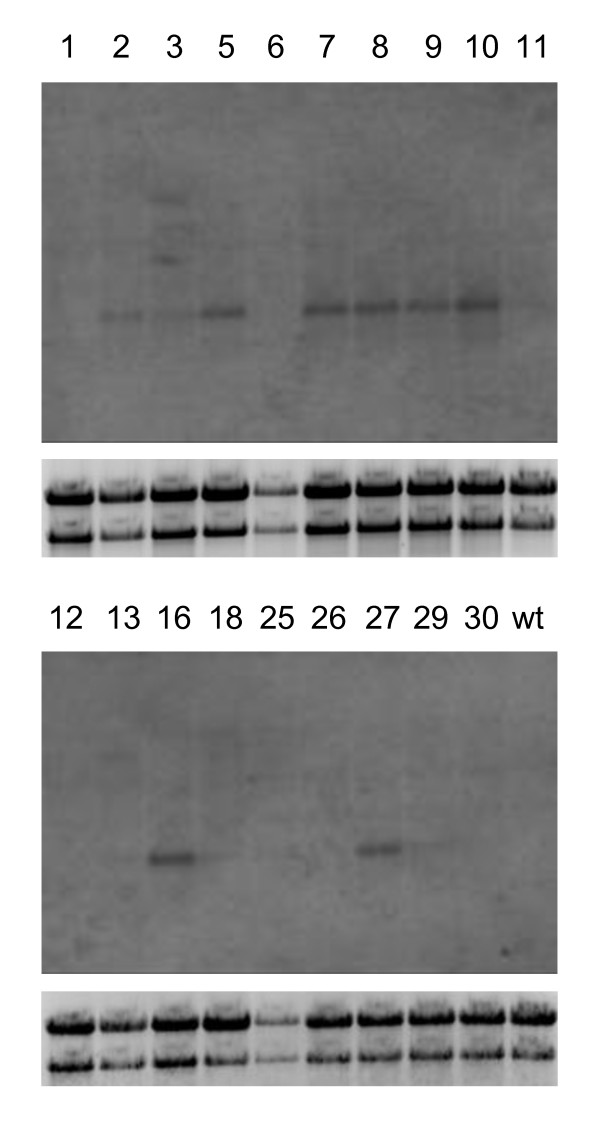
**Northern blot analysis of independent BY-2 callus clones transformed with pBin-TetR.** Approximately 10 μg of total RNA was loaded (except for #6 and #25 due to limited amounts of biomass) and hybridized with a ^32^P-labeled TetR probe. Numbers indicate the different clones. Top panels: images of the hybridized blot as visualized on a phosphorimager. Bottom panels: agarose gels stained with ethidium bromide to show intact 28 S and 18 S rRNA bands.

The strict regulation of transgene expression using a tetracycline inducible promoter requires the generation of a stable cell line expressing high levels of TetR. We therefore transformed wild-type BY-2 cells with pBinTetR [17], containing the *tet*R gene under the control of the CaMV 35 S promoter. Transformants were selected on medium supplemented with kanamycin and 19 independent callus clones were screened for *tet*R mRNA by northern blot analysis (Figure 1). Clones #8, #10 and #16 were used to establish liquid suspension cultures, all three of which grew well. We chose clone #10 for super-transformation with our inducible vIL-10 constructs and this cell line was named BY-2-TetR.

### Development of an optimized vector for tetracycline-inducible transgene expression

**Figure 2:**
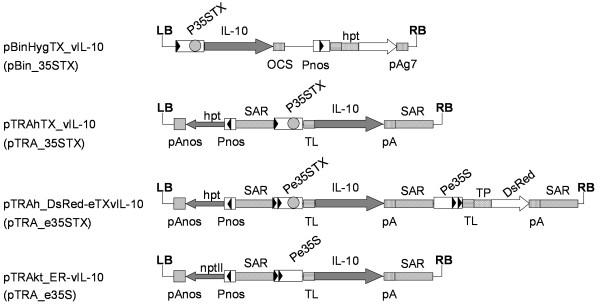
**Schematic overview of the T-DNA cassettes used for constitutive and inducible expression of vIL-10.** LB and RB, left and right border of the T-DNA; P35STX, Inducible *Cauliflower mosaic virus* 35 S promoter; Pe35S, CaMV 35 S promoter with enhancer (double arrowheads); TX, three *tet*O sites (TetR binding sites, gray circle) surrounding the TATA box; OCS, octopine synthase gene terminator; pAg7, agropine synthase gene terminator; Pnos and pAnos, nopaline synthase gene promoter and terminator; *hpt*, hygromycin phosphotransferase gene; *npt*II, neomycin phosphotransferase gene; SAR, scaffold attachment region; pA, terminator of the CaMV 35 S gene; TL, 5′ UTR of the *Tobacco etch virus*; vIL-10, viral IL-10 coding sequence with native signal peptide sequence at the 5′ end and sequences for a His_6_ tag and ER retention signal (SEKDEL) at the 3′ end; TP, plastid transit peptide from the barley granule-bound starch synthase gene; DsRed, *Discosoma* spp red fluorescent protein. The full name of the vector is shown to the side of each diagram and the vector backbone and promoter are indicated in parentheses. Not drawn to scale.

We initially introduced the sequence for the ER-targeted version of vIL-10 into pBinHygTX [17] to create pBinHygTX_vIL-10 (Figure 2). This was used to super-transform the BY-2-TetR cell line but even by this stage we were aware that the chosen vector system was suboptimal, in that the large size of pBinHygTX (12 kb, including 8.5 kb of backbone sequence) made subcloning procedures unnecessarily complicated. The preliminary induction of transgene expression in transformed callus clones was also more complex than anticipated because the induction of BY-2 cells with anhydrotetracycline (Ahtc) on solid medium as described [17] was not uniform, giving rise to heterogeneous vIL-10 yields as determined by immunodotblot or ELISA at each repetition (data not shown). We also tried an alternative strategy of inducing vIL-10 expression while the callus was in liquid medium directly after resuspension, but the addition of Ahtc resulted in extensive cell death, probably due the higher toxicity of the chemical on the already stressed cells.

We therefore decided to construct a vector carrying a tandem expression cassette comprising the tetracycline-inducible vIL-10 gene and the *Discosoma* spp. red fluorescent protein (DsRed) gene under the control of the constitutive CaMV 35 S promoter. The visible marker gene was included to facilitate the early selection of the best-performing clones based on fluorescence intensity, which correlates with the amount of protein [20]. We switched to the pTRA vector series (derivatives of pPAM [21]) because they have a smaller backbone than pBin vectors (<3 kb) and are therefore more suitable for tandem expression cassettes. Expression cassettes in the pTRA vector used are also flanked by scaffold attachment regions (SARs) from the tobacco *RB7* gene [22], and include the *Tobacco etch virus* 5′-UTR which promotes protein synthesis [23]. To maximize the expression of vIL-10 we generated a tetracycline-inducible CaMV 35 S promoter including an enhancer sequence. The final vector was named pTRAh_DsRed-eTXvIL-10 (Figure 2).

### Vector/promoter comparison

**Figure 3:**
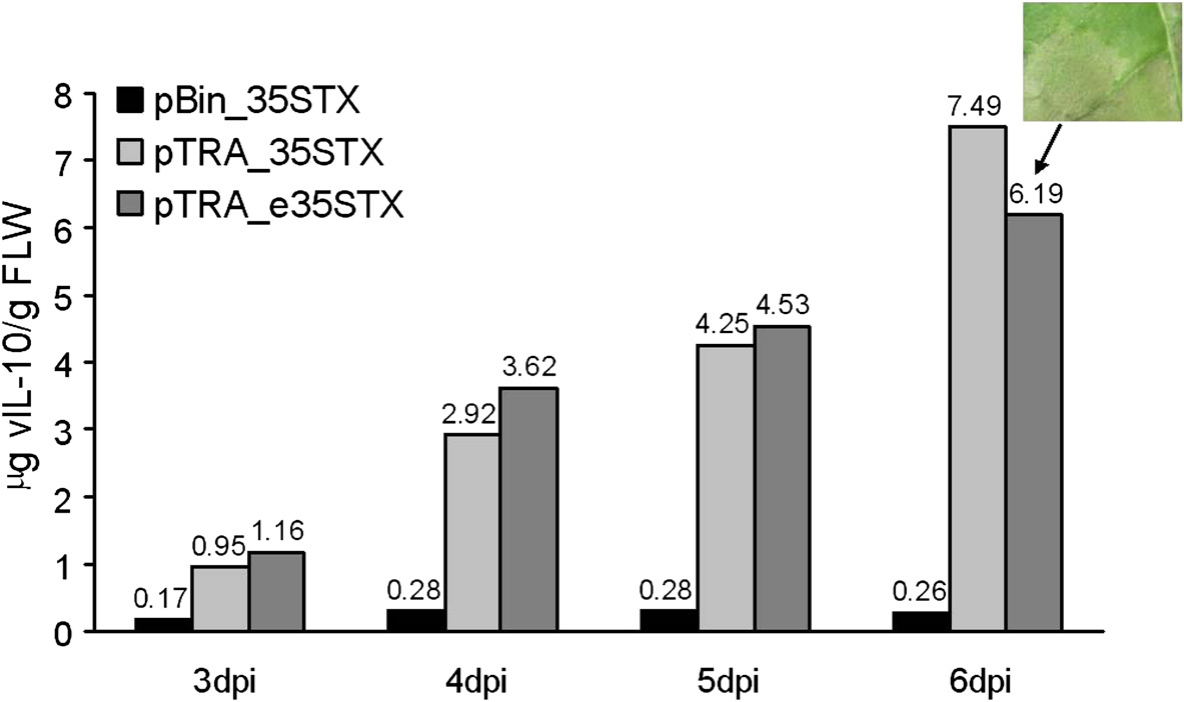
**Time course analysis of vIL-10 accumulation in wild-type tobacco leaves following infiltration with bacteria carrying different expression constructs.** ELISAs were used to determine vIL-10 levels in leaf protein extracts at 3, 4, 5 and 6 days post infiltration (dpi), expressed as μg/g FW. Vector abbreviations correspond to those in Figure 2. The insert show the phenotype of the infiltrated leaf tissue, which was mostly necrotic at 6 dpi (pTRA_e35STX).

We tested the potential of the enhanced tetracycline-inducible CaMV 35 S promoter in pTRAh_DsRed-eTXvIL-10 by transient expression in tobacco plants, using a control vector lacking the *tet*O sequences. Wild-type tobacco plants (*Nicotiana tabacum* cv. Petite Havana SR1) were individually syringe-infiltrated with *Agrobacterium tumefaciens* strains carrying one of the four different vectors available for vIL-10 expression (Figure 3). Wild-type tobacco plants do not produce TetR, so transcription from all the promoters (including the inducible ones) was constitutive, allowing the direct comparison of their efficiency. Agroinfiltration was carried out in such a way as to include all possible variations in vIL-10 expression due to the state of the plant/leaf material (two plants, three leaves per plant, and randomized leaf areas including the tip, center and base). We prepared leaf discs from the infiltrated areas of all three leaves from both plants at each time point, and pooled the samples for protein extraction and vIL-10 quantification by ELISA (Figure 3). We assumed that the onset of necrosis in the tissue infiltrated with pTRA_e35STX at 6 dpi represented high levels of recombinant protein expression (like the stunted phenotype of transgenic plants) even though detected vIL-10 in the necrotic tissue was lower due to high levels of proteolytic activity and inefficient protein extraction. We were thus able to rank the promoters/vector combinations in descending order of activity as follows: pTRA_e35STX ≥ pTRA_35STX > > pBin_35STX (Figure 3). The comparison of pTRA_35STX and pTRA_e35STX indicated that the additional enhancer sequence in pTRA_e35STX has a significant impact on recombinant protein accumulation, resulting in the appearance of necrotic lesions in the infiltrated area by 6 days post-infiltration. The pBin_35TX vector was an order of magnitude less efficient than any of the vectors based on pTRA.

### Selection of the best transgenic lines

**Figure 4:**
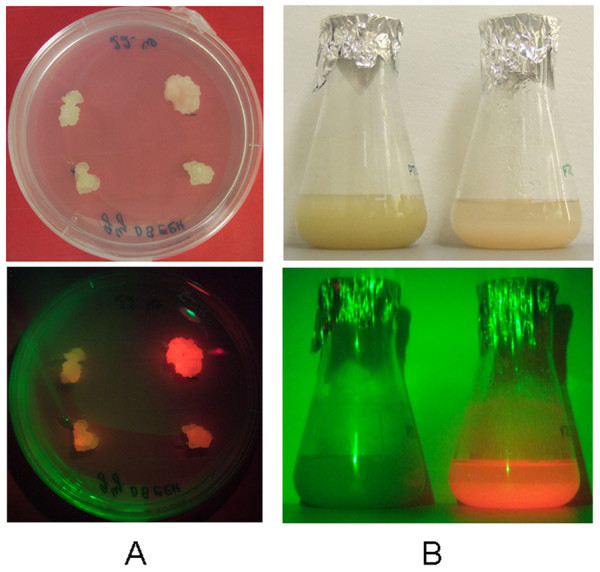
**Selection of transgenic BY-2 lines based on DsRed fluorescence.** DsRed fluorescence (lower panels) was visualized using an LCD lamp with glass fiber optics (Leica KL1500), an additional filter for green excitation (BP545/30) and a colored foil (#182, light red). (**A**) Four independent transgenic BY-2 callus clones that show macroscopically different fluorescence intensity and homogeneity. (**B**) Liquid suspension cultures of BY-2 cells, wild-type cells on the left and transgenic cells expressing DsRed on the right.

The BY-2-TetR line was super-transformed with pTRAhDsRed-eTXvIL-10 and over a hundred independent callus clones were derived by double selection on medium containing kanamycin and hygromycin. We then selected the callus clones displaying macroscopically uniform DsRed fluorescence because this is usually a good indicator of the expression levels of a linked transgene (Figure 4A). Nine callus clones representing low (#26, #28, #35) medium (#2, #5, #48) and high (#22, #36, #44) levels of DsRed fluorescence were identified by eye and transferred directly into liquid medium to establish suspension cell cultures (Fig. 4B).

**Figure 5:**
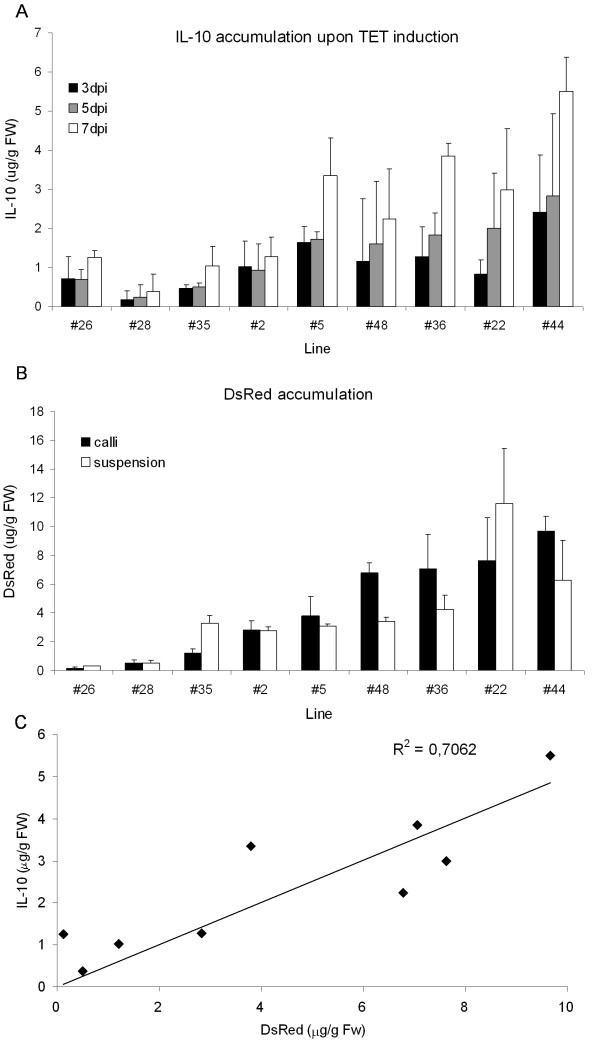
**Comparison of DsRed and vIL-10 accumulation levels.** (**A**) Cell suspension cultures from different lines were induced with 10 μM Ahtc and 2-ml aliquots were analyzed daily from 3 to 7 dpi. The accumulation of vIL-10 in cell pellet extracts was determined by ELISA. (**B**) DsRed accumulation was determined in callus and cell suspension culture extracts from each transgenic line arrayed in 96-well plates using a fluorometer. The data reported are means ± SD from three independent experiments. (**C**) Linear regression between the yields of IL-10 (in suspension cultures at 7 dpi) and DsRed (in callus) for nine clones. The correlation coefficient R^2^ is indicated inside the plot.

**Figure 6:**
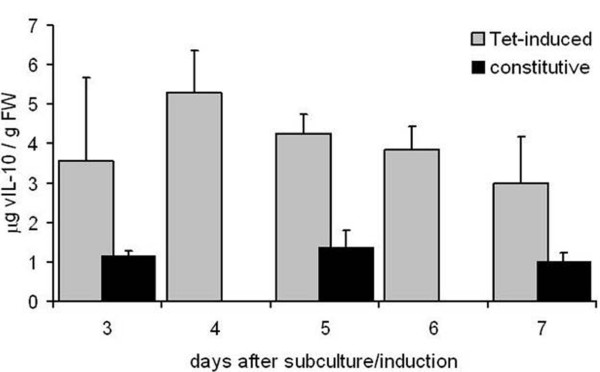
**Comparison of constitutive and inducible vIL-10 accumulation in BY-2 cell suspension cultures.** Samples were taken from cultures expressing vIL-10 constitutively (cells transformed with pTRAkt_ER-vIL-10, black bars) at 3, 5 and 7 days post subculture. Samples were taken from cultures expressing vIL-10 under induction (cells transformed with pTRAh_DsRed-eTXvIL-10, grey bars) daily from 3 to 7 days post induction. The accumulation of vIL-10 was determined in cell pellet extracts by ELISA. The data reported are expressed as μg/g FW and represent the means ± SD from three independent experiments.

As for the best two lines induction 3 days after subculture resulted in overall lower vIL-10 yields compared to induction directly at the time of subculture (e.g. 4.1 vs. 5.3 μg/g FW for line #44, Additional file 3: Figure S3), we then tested for correspondence between DsRed fluorescence intensity and vIL-10 yields by inducing all cell lines with 10 μM Ahtc at the next subculture interval. The amount of vIL-10 in cell pellet extracts was determined by ELISA at 3, 5 and 7 days post-induction (dpi) (Figure 5A). The yield of the recombinant protein increased over time, with the highest levels of vIL-10 detected 7 dpi for all lines. A similar trend in protein accumulation was observed for the constitutive expression of the same transgenes in newly-established suspension cultures whose growth in liquid medium had not been completely stabilized (data not shown). In addition to the preliminary visual evaluation of DsRed levels in the transgenic callus clones, we quantified the levels of DsRed protein in extracts from the callus clones and the derived cell suspension cultures (Figure 5B). There was excellent correspondence between the visual evaluations and accurately-determined protein levels in all cases. The amount of DsRed in the callus clones was generally slightly higher than in the corresponding suspension cells. As expected, there was also a good correlation between the amount of vIL-10 measured at 7 dpi and the level of DsRed fluorescence in the same clones (Figure 5C). Line #44 produced the highest yields of vIL-10 and was evaluated by ELISA in three independent induction experiments, sampling the culture daily from 3 to 7 dpi (Figure 6). The results were reproducible, with vIL-10 yields of up to 5.3 μg/g FW by 4 dpi, which is more than 3.5 times higher than the best-performing constitutive lines (1.4 ± 0.4 μg/g FW, Figure 6). Negligible amounts of vIL-10 were found in the non-induced control cultures of even the best-performing transgenic lines (undetectable for line #36, 0.013 μg/g FW for line #44), indicating that the BY-2-TetR cassette is tightly regulated and does not show significant leakiness.

### Impact of Ahtc on cell growth

**Figure 7:**
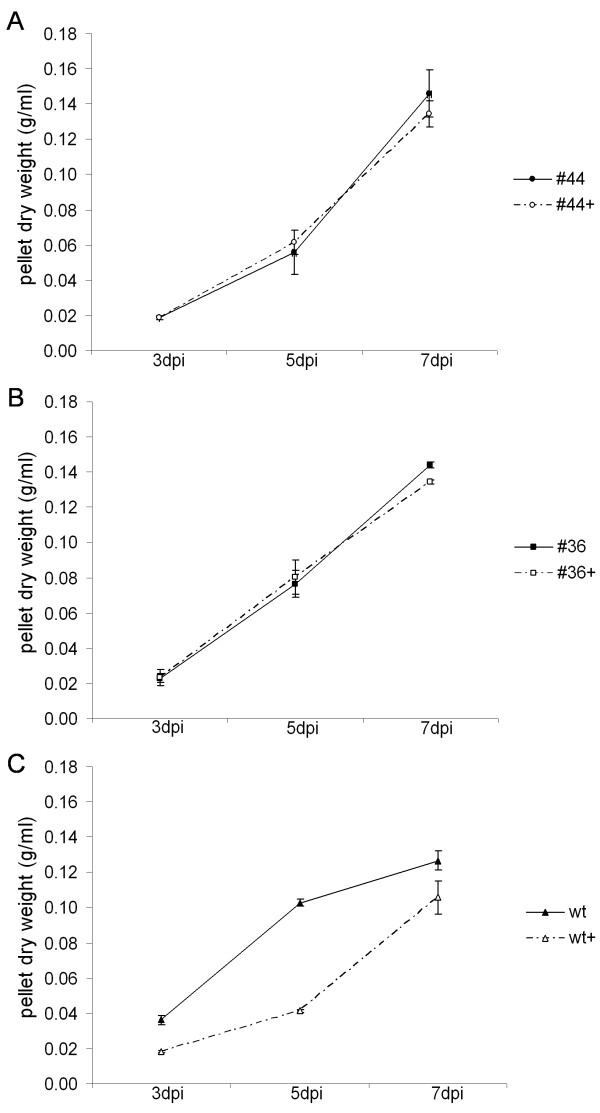
**Growth curves of BY-2 suspension cells with (+) and without 10 μM Ahtc treatment at subculture.** We withdrew 5-ml samples from 50-ml cultures of transgenic lines #44 (**A**) and #36 (**B**) or wild-type (**C**) cells at 3, 5 and 7 dpi, vacuum filtered the cells through filter paper discs (MN615) and recorded the dry biomass weight after drying at 37 °C for 48 h. The data reported are means ± SD of three independent experiments.

The growth of the transgenic lines with the highest vIL-10 levels post-induction (lines #44 and #36) was monitored under both the induced and non-induced states alongside wild-type BY-2 cells, and the biomass was determined at different intervals. As shown in Figure 7A,B, the growth curves of both transgenic lines are similar in the presence and absence of Ahtc, indicating that neither the inducer nor the accumulation of vIL-10 had a significant impact on cell growth during the investigated timeframe. In contrast, the addition of Ahtc to the wild-type cells reduced their growth significantly (Figure 7C). In the transgenic lines, binding of the TetR protein to free Ahtc probably prevents its toxic effect [24].

## Discussion

Plant cell suspension cultures are emerging as a useful platform for the manufacture of recombinant proteins [25-28]. Production lines can be generated and scaled up more quickly and with lower costs than systems based on whole plants [29]. Plant cells are also advantageous because recombinant proteins can be secreted into and recovered from the culture medium. They allow pharmaceuticals to be produced under current good manufacturing practice in bioreactor-based processes. Efficient and reliable inducible expression systems can improve the versatility of plant cell cultures still further by restricting protein expression to the most productive growth phase and preventing exposure to proteases which reduce both the quantity and the quality of the product. Inducible promoters allow transgene expression to be delayed until the culture has reached a suitable biomass in the late exponential growth phase, allowing production to be uncoupled from plant cell growth [15].

Tetracycline-specific derepressible expression [30] is a widely-characterized inducible system in plants that has been successfully applied in tobacco BY-2 cells [17]. We have developed an improved version using a tandem construct incorporating a constitutive visual marker gene that allows the rapid and straightforward selection of the best-performing transgenic lines.

The first improvement was achieved by transferring the expression cassette from a pBin vector to pTRA, resulting in a substantial increase in recombinant protein yields by transient expression in tobacco. This probably reflects the influence of the *Tobacco etch virus* 5′-UTR, which acts as a translational enhancer in plants [23]. The pTRA vector also contains flanking scaffold attachment regions (SARs), which are known to enhance transcriptional activity and mitigate silencing effects by defining a stable chromatin domain [22,31,32]. Although stable transgenic lines were not generated with the pBin vector, transient expression experiments usually provide a reliable indication of the outcome of stable transformation [33-35], thus it is reasonable to anticipate superior performance from the pTRA vector in stable transgenic lines.

The second improvement was achieved by adding a CaMV 35 S enhancer upstream of the tetracycline-inducible Triple-Op promoter. The presence of the enhancer sequence significantly increases the activity of the native CaMV 35 S promoter [36]. We anticipated similar benefits in the context of the Triple-Op promoter because it differs from the native promoter only in the presence of three *tet*O sequences surrounding the TATA box, and their presence should not hamper the synergistic interaction between the enhancer and minimal promoter.

The third and most innovative improvement was the tandem configuration of the inducible transgene and a constitutive visual marker gene, which greatly simplified the procedure for selecting the best-performing clones. Transgene expression is strongly influenced by the chromatin surrounding the integration site so tightly linked transgenes are generally subjected to the same global regulatory factors. This means that a gene encoding a fluorescent protein can be used as a surrogate marker to determine the activity of a linked transgene whose product can only be detected using as less convenient off-line assay. In this case, we found that the intensity of DsRed fluorescence was proportional to the amount of DsRed protein, which in turn was indicative of the amount of vIL-10. Although we observed a good correlation between the marker and the primary gene product, many factors could uncouple the yields of adjacent transgenes such as multiple or partial T-DNA insertions and transgene rearrangements. The DsRed gene was placed adjacent to the right-hand T-DNA border in our vector, and since the right-hand border is transferred first this configuration is more likely to produce false positives (strong fluorescence without corresponding vIL-10 accumulation) than false negatives (strong vIL-10 expression without fluorescence). Even so, the advantage of initial screening based solely on DsRed fluorescence is that second-stage screening following induction of the linked transgene is limited to a handful of promising clones, rather than tens or hundreds [37]. Should it be so desired, it is also still possible within our platform to analyze antibiotic resistant callus clones that lack DsRed fluorescence to identify potential false negative lines that produce high levels of vIL-10. We used a precise fluorometric assay to determine the levels of DsRed fluorescence in order to carry out a quantitative comparison between DsRed and vIL-10 expression and demonstrate the predictive accuracy of our platform. However, for routine deployment such a precise assay is unnecessary. The human eye cannot distinguish small variations in fluorescence but is sufficient to identify the brightest callus clones on a plate using a green fluorescent light and a red filter, allowing the rapid visual selection of promising clones without sophisticated apparatus.

Finally, the constitutively expressed visual marker can also be used as an internal control to monitor the genetic stability of selected transgenic lines during prolonged cultivation with multiple subcultures. Any loss of fluorescence would be a strong indicator of transgene disruption/loss by recombination or the onset of epigenetic gene silencing.

The combination of improvements discussed above resulted in a highly reproducible increase in the accumulation of vIL-10, 3.5 times higher than those obtained by constitutive expression in the same cell line. The inducible expression cassette was highly efficient, benefiting from a low basal expression level and a high induction ratio. We did not replenish Ahtc in the culture medium so vIL-10 accumulation peaked at 4 dpi in well-established suspension cultures and decreased thereafter (Figure 6). Further increases in yield might be achieved by optimizing the concentration and dosing method of the inducer and the timing of induction.

As well as its value in the field of molecular farming, the inducible expression cassette could also be useful for basic research by allowing the precisely-regulated overexpression or silencing of endogenous genes [15]. The accurate measurement of reporter protein fluorescence would allow the selection of cell lines with different levels of transgene expression, allowing quantitative effects to be monitored rather than the binary on/off choice that conventional assays allow. In that regard, DsRed has been used as reporter gene in BY-2 cells [38], whole plants [20,39], fungi [40] and animals [41] without any reports of adverse effects on the host.

## Conclusions

We have developed a more efficient and more convenient version of the widely used tetracycline-inducible expression system, and have used it to increase the accumulation of the toxic cytokine vIL-10 in BY-2 cell suspension cultures. The new platform increases both the quality and the quantity of the final product, and allows the high-level production of recombinant proteins that would affect the growth and vitality of cells when expressed constitutively. Although we demonstrated the platform using BY-2 cells to produce vIL-10, the components can be transferred easily to any other host cell, allowing the platform to be used both for molecular farming and basic research.

## Methods

### Constructs

Vectors pTRAkt_ER-vIL-10 and pTRAkt_ER-mIL-10 have already been described [14]. Vectors pBinHygTx, pBinTetR and pUC-TetR were kindly provided by Prof. Dr. Christiane Gatz (Georg-August-Universität Göttingen, D). Vector pBinHygTx_vIL-10 was generated by amplifying the vIL-10 coding sequence from vector pTRAkt_ER-vIL-10 to introduce *Kpn*I and *Xba*I restriction sites at the 5′ and 3′ termini, using primers KpnI_vIL-10_for (5′-CGG GGT ACC ATG GAG CGA AGG TTA GTG GTC-3′) and vIL-10-KDEL_rev (5′-GCT CTA GAC GGT TTA GAG CTC ATC TTT CTC AGA CCT GGC TTT AAT TGT CAT GTA TGC-3′). The PCR product was then transferred as a *Kpn*I-*Xba*I fragment into pBinHygTx, linearized with the same enzymes.

To generate the other vectors, a 562-bp fragment comprising the CaMV 35 S promoter with three *tet*O sites was amplified from vector pBinHygTx using primers 35 S-TOP5 (5′-CAA TGG CGC GCC AAA GAT TCA AAT AGA G-3′) and 35 S-TOP3 (5′-TCC CCG AAT TCC GTT AAC TC-3′). The product was digested with *Asc*I and *Eco*RI (546 bp) or *Acc*I and *Eco*RI (414 bp). The *Asc*I-*Eco*RI fragment was introduced, together with a 693-bp *Eco*RI-*Xba*I fragment from pTRAkt_ER-vIL-10 containing the *Tobacco etch virus* 5′-UTR and the coding sequence of vIL-10, into pTRAh (pTRA vector containing a hygromycin phosphotransferase marker) digested with *Asc*I and *Xba*I to create pTRAhTX_vIL-10. The *Acc*I-*Eco*RI fragment was introduced, together with the 693-bp *Eco*RI-*Xba*I vIL-10 fragment into pTRAh digested with *Acc*I and *Xba*I to generate pTRAheTX_vIL-10. The pTRAh_DsRed-eTXvIL-10 vector was generated by transferring the 4331-bp *Sac*I-*Pme*I fragment from pTRAk-2F5ER-Ds [29], containing a constitutively-expressed and plastid-targeted DsRed gene, into pTRAheTX_vIL-10.

### Cell culture maintenance, transformation and biomass determination

Tobacco (*Nicotiana tabacum* cv. Bright Yellow 2; BY-2) suspension cells were cultivated in liquid medium (3% (w/v) sucrose, 4.4 g/L Murashige and Skoog (MS) salts with minimum organics, 0.4 mg/l thiamine, 0.2 mg/L 2,4-dichlorophenoxyacetic acid, 200 mg/L KH_2_PO_4_, pH 5.8) at 26 °C in the dark, shaking at 180 rpm. Suspension cells were subcultured weekly using a 5% (v/v) inoculum. Callus cultures were maintained on solid medium (MS medium plus 0.8% (w/v) agar) in the dark and transferred as necessary.

BY-2 cells were transformed by co-cultivation with *Agrobacterium tumefaciens* (strain GV3101:pMP90RK for the pTRA based vectors, and GV2260 for pBin based vectors) by adding 150 μl of bacterial suspension (OD_600_ ~1) to 3 ml of a 3-day-old suspension cell culture supplemented with 200 μM acetosyringone. After 3 days of co-incubation at 26 °C, cells were plated onto solid medium containing 200 mg/L cefotaxime and either 100 mg/L kanamycin (for pBinTetR, pTRAkt_ER-mIL-10 and pTRAkt_ER-vIL-10) or 100 mg/L kanamycin and 50 mg/L hygromycin (for the super-transformation of BY-2-TetR with pBinHygTX_vIL-10, pTRAhDsRed-eTXvIL-10 and pTRAh-TXvIL-10). After incubation for 3–4 weeks at 26 °C, antibiotic-resistant callus clones were screened for DsRed fluorescence and transferred to fresh plates. The expanded clones were used to establish cell suspension cultures. The cultures were induced by adding 10 μM Ahtc (Sigma-Aldrich, St. Louis, Mo, USA) to the liquid medium during subculture.

Biomass dry weight was determined by vacuum filtering 5 ml of each culture through filter paper (MN615, Macherey & Nagel, Düren, Germany), and drying the cells at 37 °C for 48 h.

### Northern blot analysis

Northern blot analysis was carried out as previously described [42]. Briefly, total RNA was prepared from transgenic callus tissue using the NucleoSpin RNA plant kit (Macherey & Nagel). We loaded 10 μg of total RNA onto a denaturing agarose gel and blotted the separated nucleic acids onto a positively charged nylon membrane, which was probed with a ^32^P-labeled 1.4-kb *Eco*RI/*Hin*dIII fragment released from pUC-TetR.

### Isolation of total soluble proteins and IL-10 quantification

Approximately 200 mg of callus tissue or cell pellet biomass prepared from the liquid culture by centrifugation for 10 min at 16,000 g, 4 °C were weighed in a 2-ml plastic tube, and sonicated on ice for 1 min (9 x 10% cycle, 35% power) with two volumes of BY-2 extraction buffer (PBS pH 7.4, 0.05% (v/v) Tween-20, 5 mM EDTA, pH 8.0, (v/v) freshly added DMSO). The clear supernatant was separated from cell debris by centrifugation at 16,000 x g for 30 min at 4 °C, and the total soluble protein content was determined using the Roti^®^Quant reagent (Roth, Karlsruhe, Germany). Murine and viral IL-10 levels were quantified by sandwich ELISA [14]. The detection limit of IL-10 in ELISA is ~10 pg/ml for the viral and ~50 pg/ml for the murine IL-10, respectively.

### Visualization and quantitation of DsRed fluorescence

DsRed fluorescence was visualized in transiently infiltrated tobacco leaves, stably transformed BY-2 callus clones and cell suspension cultures using an LCD lamp with glass fiber optics (KL1500, Leica, Wetzlar, Germany), an additional filter for green excitation (Leica BP545/30) and a colored foil (# 182 light red, Lee Filters, Andover, UK). DsRed fluorescence in cell extracts was quantified in 96-well plates using a Synergy HT microtiterplate reader (Bio-TEK, Bad Friedrichshall, Germany) against a calibration curve prepared from 50 to 0.04 μg/ml solutions of home-made affinity-purified DsRed stock.

## Competing interest

The authors declare that they have no competing interest.

## Authors’ contributions

LB participated in the design of the study, performed the induction experiments and IL-10 and DsRed measurements, analyzed the data and wrote the manuscript. TR conceived the cloning strategy and generated the new vectors, performed the growth curves analysis and helped drafting the manuscript. AS selected and characterized the repressor line and critically revised the manuscript. FS generated all transgenic lines and helped interpreting the results. MP and SS conceived the study, participated in its design and coordination, interpretation of data and critical reading of the manuscript. All authors read and approved the final manuscript.

## Supplementary Material

Additional file 1 **Figure S1. Phenotype of transgenic tobacco (*Nicotiana tabacum*cv. Petite Havana SR1) plants stably transformed with the pTRAkt_ER-vIL-10 construct (constitutive vIL-10 expression).** Analysis of vIL-10 accumulation levels in transgenic tobacco plants revealed a striking correspondence between the stunted phenotype and the amounts of recombinant protein detected in the leaf tissue. In this figure, three T_1_ plants of the same age and grown under the same conditions, representative of the range of phenotypic alterations observed, are shown, and the corresponding accumulation levels of vIL-10 measured by ELISA in the leaf extract are reported below each plant (A). No differences could be detected regarding the appearance or the growth rate of the transgenic plant accumulating vIL-10 to the lowest level and the wild type (not shown). (B) Enlargement of the dwarf plant in (A), which accumulates vIL-10 at the highest levels and displays the strongest phenotype observed. Note that, besides the stunted appearance, with shorter internodes and smaller leaves, this plant is still in a vegetative state, while the least-expressing normal-looking plant of the same age is already flowering. (C) Side view of the same transgenic plants shown in (A), underlining the enormous difference in growth and development between the three expressers. Click here for file

Additional file 2 **Figure S2. Biochemical characterization of BY-2-produced viral and murine IL-10.** (A). Immunoblot analysis of recombinant vIL-10 and mIL-10 purified from transgenic BY-2 cells confirmed the correct molecular weight of 19 and 21 kDa, respectively. (B). The purified proteins were digested with trypsin and the peptides subjected to LC-MS analysis. As expected, vIL-10 was not glycosylated (not shown). The murine IL-10 was confirmed to be glycosylated and the N-glycans found were only of the oligo-mannose-type, as expected for a protein retained in the ER. The spectrum of the glycosylated peptide is shown. See http://www.proglycan.com for an explanation of N-glycan abbreviations. For the methods refer to Bortesi et al. 2009. Click here for file

Additional file 3 **Figure S3. Time course analysis of vIL-10 accumulation in transgenic cultures induced 3 days after subculture.** Cell suspension cultures the two most promising lines were induced with 10 μM Ahtc 3 days after subculture and 2-ml aliquots were analyzed daily from 1 to 4 dpi. The accumulation of vIL-10 in cell pellet extracts was determined by ELISA. The data reported are expressed as μg/g FW and represent the means ± SD from three independent experiments. Click here for file

## References

[B1] Schiermeyer A Schillberg S Kempken F, Jung C Pharmaceuticals Genetic modification of plants: agriculture, horticulture and forestry 2010 Springer, Berlin Heidelberg 221 235

[B2] Faye L Gomord V Success stories in molecular farming-a brief overview Plant Biotechnol J 2010 8 5 525 528 10.1111/j.1467-7652.2010.00521.x 20500680

[B3] Bock R Warzecha H Solar-powered factories for new vaccines and antibiotics Trends Biotechnol 2010 28 5 246 252 10.1016/j.tibtech.2010.01.006 20207435

[B4] Peters J Stoger E Transgenic crops for the production of recombinant vaccines and anti-microbial antibodies Hum Vaccin 2011 7 3 10.4161/hv.7.3.14303 21346415

[B5] Tremblay R Wang D Jevnikar AM Ma S Tobacco, a highly efficient green bioreactor for production of therapeutic proteins Biotechnol Adv 2010 28 2 214 221 10.1016/j.biotechadv.2009.11.008 19961918 PMC7132750

[B6] Obembe OO Popoola JO Leelavathi S Reddy SV Advances in plant molecular farming Biotechnol Adv 2011 29 2 210 222 10.1016/j.biotechadv.2010.11.004 21115109

[B7] Zhong J Plant cells (Advances in biochemical engineering-biotechnology) 2001 Springer, Berlin Heidelberg

[B8] Schiermeyer A Schinkel H Apel S Fischer R Schillberg S Production of Desmodus rotundus salivary plasminogen activator alpha1 (DSPAalpha1) in tobacco is hampered by proteolysis Biotechnol Bioeng 2005 89 7 848 858 10.1002/bit.20410 15685597

[B9] Huang TK Plesha MA McDonald KA Semicontinuous bioreactor production of a recombinant human therapeutic protein using a chemically inducible viral amplicon expression system in transgenic plant cell suspension cultures Biotechnol Bioeng 2010 106 3 408 421 20198659 10.1002/bit.22713

[B10] Sorrentino A Schillberg S Fischer R Porta R Mariniello L Molecular farming of human tissue transglutaminase in tobacco plants Amino Acids 2009 36 4 765 772 10.1007/s00726-008-0132-8 18594943

[B11] Avesani L Vitale A Pedrazzini E Devirgilio M Pompa A Barbante A Gecchele E Dominici P Morandini F Brozzetti A Falorni A Pezzotti M Recombinant human GAD65 accumulates to high levels in transgenic tobacco plants when expressed as an enzymatically inactive mutant Plant Biotechnol J 2010 8 8 862 872 10.1111/j.1467-7652.2010.00514.x 20374524

[B12] Zhou F Badillo-Corona JA Karcher D Gonzalez-Rabade N Piepenburg K Borchers AM Maloney AP Kavanagh TA Gray JC Bock R High-level expression of human immunodeficiency virus antigens from the tobacco and tomato plastid genomes Plant Biotechnol J 2008 6 9 897 913 10.1111/j.1467-7652.2008.00356.x 19548344

[B13] Ehsani P Meunier A Nato F Jafari A Nato A Lafaye P Expression of anti human IL-4 and IL-6 scFvs in transgenic tobacco plants Plant Mol Biol 2003 52 1 17 29 10.1023/A:1023902407855 12825686

[B14] Bortesi L Rossato M Schuster F Raven N Stadlmann J Avesani L Falorni A Bazzoni F Bock R Schillberg S Pezzotti M Viral and murine interleukin-10 are correctly processed and retain their biological activity when produced in tobacco BMC Biotechnol 2009 9 22 10.1186/1472-6750-9-22 19298643 PMC2667500

[B15] Corrado G Karali M Inducible gene expression systems and plant biotechnology Biotechnol Adv 2009 27 6 733 743 10.1016/j.biotechadv.2009.05.006 19460424

[B16] Padidam M Chemically regulated gene expression in plants Curr Opin Plant Biol 2003 6 2 169 177 10.1016/S1369-5266(03)00005-0 12667875

[B17] David KM Perrot-Rechenmann C Characterization of a tobacco Bright Yellow 2 cell line expressing the tetracycline repressor at a high level for strict regulation of transgene expression Plant Physiol 2001 125 4 1548 1553 10.1104/pp.125.4.1548 11299335 PMC1539379

[B18] Gatz C Lenk I Promoters that respond to chemical inducers Trends Plant Sci 1998 3 9 352 358 10.1016/S1360-1385(98)01287-4

[B19] David KM Couch D Braun N Brown S Grosclaude J Perrot-Rechenmann C The auxin-binding protein 1 is essential for the control of cell cycle Plant J 2007 50 2 197 206 10.1111/j.1365-313X.2007.03038.x 17376160

[B20] Jach G Binot E Frings S Luxa K Schell J Use of red fluorescent protein from Discosoma sp. (dsRED) as a reporter for plant gene expression Plant J 2001 28 4 483 491 11737785 10.1046/j.1365-313x.2001.01153.x

[B21] Rademacher T Hausler RE Hirsch HJ Zhang L Lipka V Weier D Kreuzaler F Peterhansel C An engineered phosphoenolpyruvate carboxylase redirects carbon and nitrogen flow in transgenic potato plants Plant J 2002 32 1 25 39 10.1046/j.1365-313X.2002.01397.x 12366798

[B22] Allen GC Hall G Michalowski S Newman W Spiker S Weissinger AK Thompson WF High-level transgene expression in plant cells: effects of a strong scaffold attachment region from tobacco Plant Cell 1996 8 5 899 913 8672887 10.1105/tpc.8.5.899 PMC161147

[B23] Carrington JC Freed DD Cap-inependent enhancement of translation by a plant potyvirus 5′ nontranslated region J Virol 1990 64 4 1590 1597 2319646 10.1128/jvi.64.4.1590-1597.1990 PMC249294

[B24] Bowman SM Drzewiecki KE Mojica ER Zielinski AM Siegel A Aga DS Berry JO Toxicity and reductions in intracellular calcium levels following uptake of a tetracycline antibiotic in Arabidopsis Environ Sci Technol 2011 45 20 8958 8964 10.1021/es200863j 21882870

[B25] Plasson C Michel R Lienard D Saint-Jore-Dupas C Sourrouille C de March G Gomord V Production of recombinant proteins in suspension-cultured plant cells Methods Mol Biol 2009 483 145 161 10.1007/978-1-59745-407-0_9 19183898

[B26] Xu J Ge X Dolan MC Towards high-yield production of pharmaceutical proteins with plant cell suspension cultures Biotechnol Adv 2011 29 3 278 299 10.1016/j.biotechadv.2011.01.002 21236330

[B27] Weathers PJ Towler MJ Xu J Bench to batch: advances in plant cell culture for producing useful products Appl Microbiol Biotechnol 2010 85 5 1339 1351 10.1007/s00253-009-2354-4 19956945

[B28] Hellwig S Drossard J Twyman RM Fischer R Plant cell cultures for the production of recombinant proteins Nat Biotechnol 2004 22 11 1415 1422 10.1038/nbt1027 15529167

[B29] Sack M Paetz A Kunert R Bomble M Hesse F Stiegler G Fischer R Katinger H Stoeger E Rademacher T Functional analysis of the broadly neutralizing human anti-HIV-1 antibody 2 F5 produced in transgenic BY-2 suspension cultures FASEB J 2007 21 8 1655 1664 10.1096/fj.06-5863com 17327362

[B30] Gatz C Quail PH Tn10-encoded tet repressor can regulate an operator-containing plant promoter Proc Natl Acad Sci USA 1988 85 5 1394 1397 10.1073/pnas.85.5.1394 2830617 PMC279777

[B31] Iglesias VA Moscone EA Papp I Neuhuber F Michalowski S Phelan T Spiker S Matzke M Matzke AJ Molecular and cytogenetic analyses of stably and unstably expressed transgene loci in tobacco Plant Cell 1997 9 8 1251 1264 9286104 10.1105/tpc.9.8.1251 PMC156995

[B32] Halweg C Thompson WF Spiker S The rb7 matrix attachment region increases the likelihood and magnitude of transgene expression in tobacco cells: a flow cytometric study Plant Cell 2005 17 2 418 429 10.1105/tpc.104.028100 15659622 PMC548816

[B33] Stoger E Vaquero C Torres E Sack M Nicholson L Drossard J Williams S Keen D Perrin Y Christou P Fischer R Cereal crops as viable production and storage systems for pharmaceutical scFv antibodies Plant Mol Biol 2000 42 4 583 590 10.1023/A:1006301519427 10809004

[B34] Torres E Vaquero C Nicholson L Sack M Stoger E Drossard J Christou P Fischer R Perrin Y Rice cell culture as an alternative production system for functional diagnostic and therapeutic antibodies Transgenic Res 1999 8 6 441 449 10.1023/A:1008969031219 10767987

[B35] Vaquero C Sack M Schuster F Finnern R Drossard J Schumann D Reimann A Fischer R A carcinoembryonic antigen-specific diabody produced in tobacco FASEB J 2002 16 3 408 410 11790722 10.1096/fj.01-0363fje

[B36] Kay R Chan A Daly M McPherson J Duplication of CaMV 35 S Promoter Sequences Creates a Strong Enhancer for Plant Genes Science 1987 236 4806 1299 1302 10.1126/science.236.4806.1299 17770331

[B37] Huang J Wu L Yalda D Adkins Y Kelleher SL Crane M Lonnerdal B Rodriguez RL Huang N Expression of functional recombinant human lysozyme in transgenic rice cell culture Transgenic Res 2002 11 3 229 239 10.1023/A:1015663706259 12113455

[B38] Holland T Sack M Rademacher T Schmale K Altmann F Stadlmann J Fischer R Hellwig S Optimal nitrogen supply as a key to increased and sustained production of a monoclonal full-size antibody in BY-2 suspension culture Biotechnol Bioeng 2010 107 2 278 289 10.1002/bit.22800 20506104

[B39] Rademacher T Sack M Arcalis E Stadlmann J Balzer S Altmann F Quendler H Stiegler G Kunert R Fischer R Stoger E Recombinant antibody 2 G12 produced in maize endosperm efficiently neutralizes HIV-1 and contains predominantly single-GlcNAc N-glycans Plant Biotechnol J 2008 6 2 189 201 10.1111/j.1467-7652.2007.00306.x 17979949

[B40] Janus D Hoff B Hofmann E Kuck U An efficient fungal RNA-silencing system using the DsRed reporter gene Appl Environ Microbiol 2007 73 3 962 970 10.1128/AEM.02127-06 17142377 PMC1800780

[B41] Wittamer V Bertrand JY Gutschow PW Traver D Characterization of the mononuclear phagocyte system in zebrafish Blood 2011 117 26 7126 7135 10.1182/blood-2010-11-321448 21406720

[B42] Schiermeyer A Hartenstein H Mandal MK Otte B Wahner V Schillberg S A membrane-bound matrix-metalloproteinase from Nicotiana-tabacum cv. BY-2 is induced by bacterial pathogens BMC Plant Biol 2009 9 83 10.1186/1471-2229-9-83 19563670 PMC2715019

