# Supplementary material for: Development of an optimized tetracycline-inducible expression system to increase the accumulation of interleukin-10 in tobacco BY-2 suspension cells
Source: BMC Biotechnol. 2012 Jul 11;12:40. doi: 10.1186/1472-6750-12-40 (PMC3410776; doi:10.1186/1472-6750-12-40)
Supplement: Additional file 1 — Figure S1. Phenotype of transgenic tobacco (Nicotiana tabacumcv. Petite Havana SR1) plants stably transformed with the pTRAkt_ER-vIL-10 construct (constitutive vIL-10 expression). Analysis of vIL-10 accumulation levels in transgenic tobacco plants revealed a striking correspondence between the stunted phenotype and the amounts of recombinant protein detected in the leaf tissue. In this figure, three T1 plants of the same age and grown under the same conditions, representative of the range of phenotypic alterations observed, are shown, and the corresponding accumulation levels of vIL-10 measured by ELISA in the leaf extract are reported below each plant (A). No differences could be detected regarding the appearance or the growth rate of the transgenic plant accumulating vIL-10 to the lowest level and the wild type (not shown). (B) Enlargement of the dwarf plant in (A), which accumulates vIL-10 at the highest levels and displays the strongest phenotype observed. Note that, besides the stunted appearance, with shorter internodes and smaller leaves, this plant is still in a vegetative state, while the least-expressing normal-looking plant of the same age is already flowering. (C) Side view of the same transgenic plants shown in (A), underlining the enormous difference in growth and development between the three expressers. [file 1472-6750-12-40-S1.pdf]

Additional material

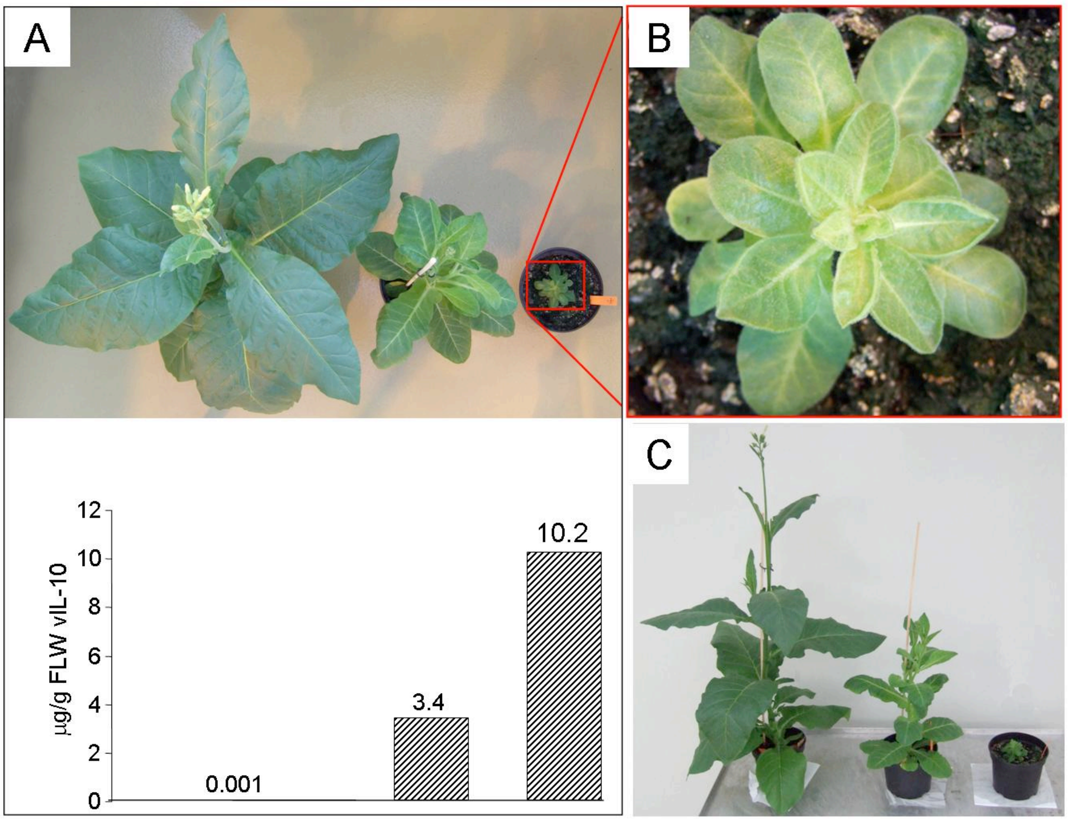

Additional Figure 1: Phenotype of transgenic tobacco (*Nicotiana tabacum* cv. Petite Havana SR1) plants stably transformed with the pTRAkt_ER-vIL-10 construct (constitutive vIL-10 expression).

Analysis of vIL-10 accumulation levels in transgenic tobacco plants revealed a striking correspondence between the stunted phenotype and the amounts of recombinant protein detected in the leaf tissue. In this figure, three T_1 plants of the same age and grown under the same conditions, representative of the range of phenotypic alterations observed, are shown, and the corresponding accumulation levels of vIL-10 measured by ELISA in the leaf extract are reported below each plant (**A**). No differences could be detected regarding the appearance or the growth rate of the transgenic plant accumulating vIL-10 to the lowest level and the wild type (not shown). (**B**) Enlargement of the dwarf plant in (A), which accumulates vIL-10 at the highest levels and displays the strongest phenotype observed. Note that, besides the stunted

appearance, with shorter internodes and smaller leaves, this plant is still in a vegetative state, while the least-expressing normal-looking plant of the same age is already flowering. (**C**) Side view of the same transgenic plants shown in (A), underlining the enormous difference in growth and development between the three expressers.
