# Supplementary figures and images for: Development of an optimized tetracycline-inducible expression system to increase the accumulation of interleukin-10 in tobacco BY-2 suspension cells
Source: BMC Biotechnol. 2012 Jul 11;12:40. doi: 10.1186/1472-6750-12-40 (PMC3410776; doi:10.1186/1472-6750-12-40)

## Slide 1
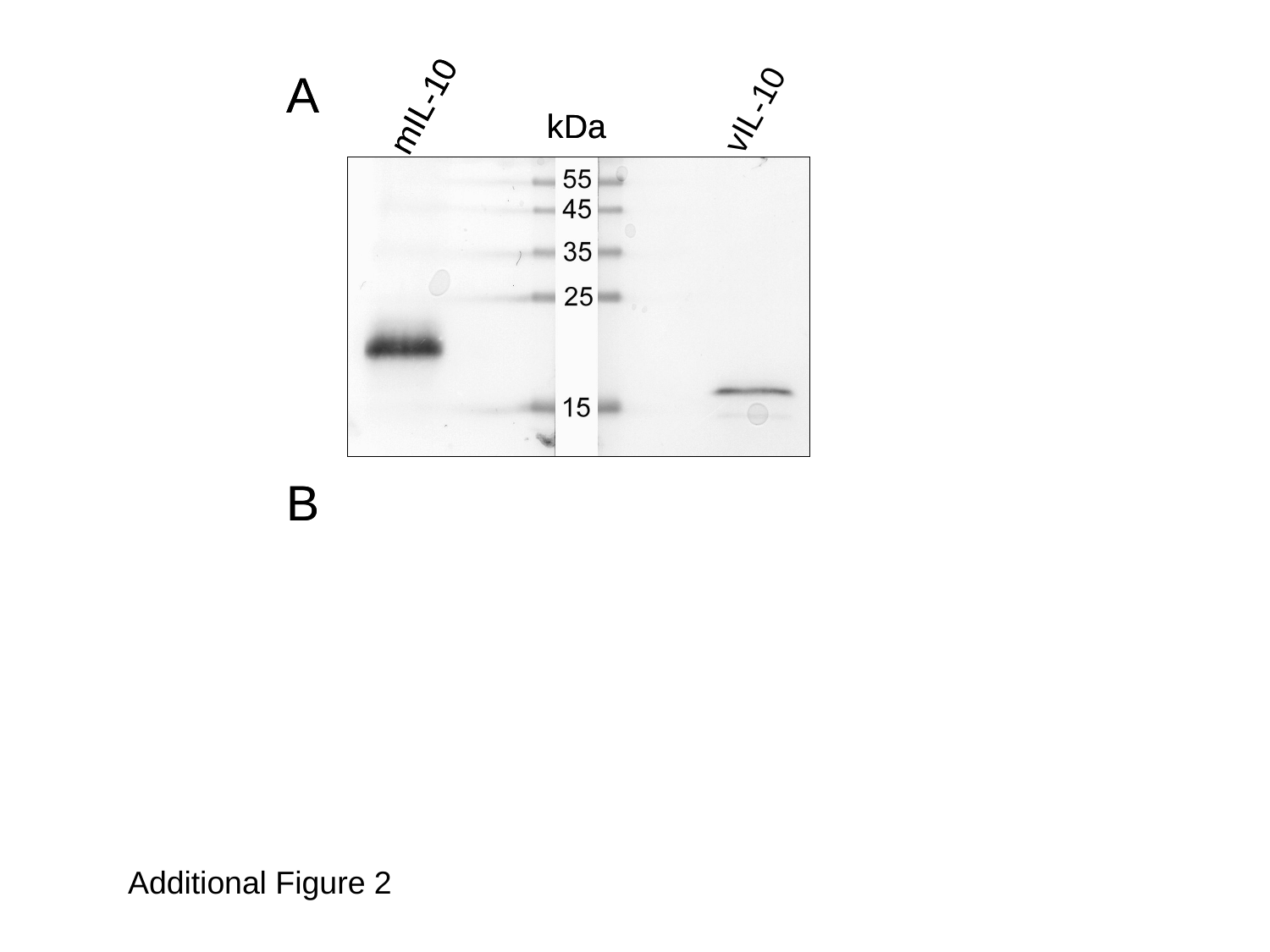

Additional Figure 2

Supplement: Additional file 2 — Figure S2. Biochemical characterization of BY-2-produced viral and murine IL-10. (A). Immunoblot analysis of recombinant vIL-10 and mIL-10 purified from transgenic BY-2 cells confirmed the correct molecular weight of 19 and 21 kDa, respectively. (B). The purified proteins were digested with trypsin and the peptides subjected to LC-MS analysis. As expected, vIL-10 was not glycosylated (not shown). The murine IL-10 was confirmed to be glycosylated and the N-glycans found were only of the oligo-mannose-type, as expected for a protein retained in the ER. The spectrum of the glycosylated peptide is shown. See http://www.proglycan.com for an explanation of N-glycan abbreviations. For the methods refer to Bortesi et al. 2009. [file 1472-6750-12-40-S2.ppt]

## Slide 1
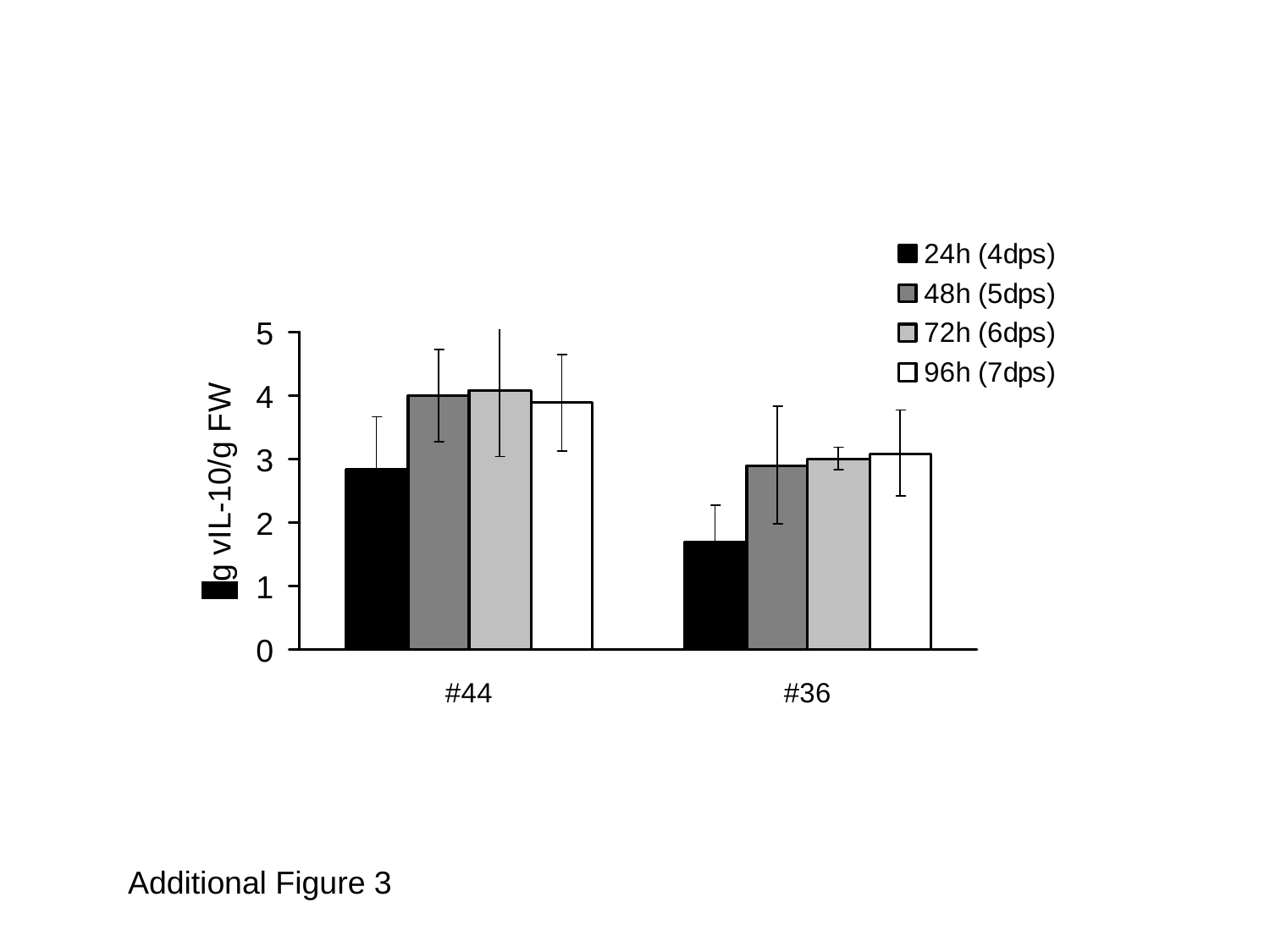

Additional Figure 3

Supplement: Additional file 3 — Figure S3. Time course analysis of vIL-10 accumulation in transgenic cultures induced 3 days after subculture. Cell suspension cultures the two most promising lines were induced with 10 μM Ahtc 3 days after subculture and 2-ml aliquots were analyzed daily from 1 to 4 dpi. The accumulation of vIL-10 in cell pellet extracts was determined by ELISA. The data reported are expressed as μg/g FW and represent the means ± SD from three independent experiments. [file 1472-6750-12-40-S3.ppt]
